# What Women With Disabilities Write in Personal Blogs About Pregnancy and Early Motherhood: Qualitative Analysis of Blogs

**DOI:** 10.2196/12355

**Published:** 2019-03-14

**Authors:** Michelle L Litchman, MJ Tran, Susan E Dearden, Jia-Wen Guo, Sara E Simonsen, Lauren Clark

**Affiliations:** 1 College of Nursing University of Utah Salt Lake City, UT United States

**Keywords:** disabled persons, pregnancy, blog, women’s health, parenting, mothers, spinal cord injury, autism, traumatic brain injury

## Abstract

**Background:**

More than 1 in 10 women of reproductive age identify as having some type of disability. Most of these women are able to become pregnant and have similar desires for motherhood as women without disability. Women with disability, however, face greater stigma and stereotyping, additional risk factors, and may be less likely to receive adequate reproductive health care compared with their peers without disability. More and more individuals, including those with disability, are utilizing the internet to seek information and peer support. Blogs are one source of peer-to-peer social media engagement that may provide a forum for women with disability to both share and obtain peer-to-peer information and support. Nevertheless, it is not clear what content about reproductive health and pregnancy and/or motherhood is featured in personal blogs authored by women with spinal cord injury (SCI), traumatic brain injury (TBI), spina bifida, and autism.

**Objective:**

The objective of this study was twofold: (1) to examine the information being shared in blogs by women with 4 types of disabilities, namely, SCI, TBI, spina bifida, and autism, about reproductive health, disability, health care, pregnancy, and motherhood; and (2) to classify the content of reproductive health experiences addressed by bloggers to better understand what they viewed as important.

**Methods:**

Personal blogs were identified by searching Google with keywords related to disabilities, *SCI*, *TBI*, *spina bifida*, and *autism*, and a variety of keywords related to reproductive health. The first 10 pages of each database search in Google, based on the relevance of the search terms, were reviewed and all blogs in these pages were included. Blog inclusion criteria were as follows: (1) written by a woman or care partner (ie, parent or spouse) of a woman with a self-identified diagnosis of SCI, TBI, spina bifida, or autism; (2) focused on the personal experience of health and health care during the prepregnancy, prenatal, antepartum, intrapartum, and/or postpartum periods; (3) written in English; and (4) published between 2013 and 2017. A descriptive and thematic qualitative analysis of blogs and corresponding comments was facilitated with NVivo software and matrix analysis.

**Results:**

Our search strategy identified 125 blogs that met all the inclusion criteria; no blogs written by women with spina bifida were identified. We identified 4 reproductive health themes featured in the blog of women with disabilities: (1) (in)accessible motherhood, (2) (un)supportive others, (3) different, but not different, and (4) society questioning motherhood.

**Conclusions:**

This analysis of personal blogs about pregnancy and health care written by women with SCI, TBI, and autism provides a glimpse into their experiences. The challenges faced by these women and the adaptations they made to successfully navigate pregnancy and early motherhood provide insights that can be used to shape future research.

## Introduction

### Background

Among women of reproductive age, approximately 12% identify as having some type of disability [1]. Most women with disabilities are able to become pregnant and fertility rates are similar among those with and without disability [2,3]. According to a large study of 10,718 women who responded to the US National Survey of Family Growth from 2006 to 2010, those with and without disability have similar attitudes about motherhood [4]. In addition, childless women are equally likely to want a child and intend to have a child, whether or not they have a disability [4].

Pregnancy and early motherhood for women with disabilities carry increased risk for poor maternal and neonatal outcomes [5-14]. The complicated web of social and biological disease risks accrues synergistically. Using a syndemic framework [15,16], the interplay of social, biological, and structural factors that place women at risk for compromised reproductive health outcomes mutually informs and reinforces disadvantage. Compared with women without disability, women with disability encounter challenges that heighten their reproductive risks. Risks include physical and emotional abuse, being overweight or obese, and decreased physical activity, inadequate emotional support, smaller social networks [17,18], substance abuse [19-24], higher levels of stress [25-27], depression and anxiety [28,29], and special needs related to their care [30]. Negative synergies ensue. *Syndemic suffering* [31] refers to the depletion of personal and material resources as individuals and their social networks respond to interactive disability-specific biological factors and social disadvantages. When women experience chronic stigma and increasing ill-health in a negative cycle of interactive disadvantage, disability scholars coin this a trajectory of *recursive cascades* [22].

The measurable outcomes of syndemic relationships between social circumstances, disability, and women’s reproductive outcomes are numerous and most often negative. Pregnant women with disabilities may be at higher risk for stillbirth, preterm birth, low birth weight, fetal growth restriction, and cesarean delivery, as well as medical complications such as venous thromboembolism and recurrent urinary tract infections [5-14]. Women with disability may also be at higher risk for postpartum depression [6] and less likely to breastfeed their infants than women without disability [7]. During pregnancy, women with disability are more likely to visit an emergency department, be admitted to the hospital, experience medical complications, and be readmitted within 3 months of their delivery [32,33] and are less likely to receive early prenatal care [33].

Enacting disability as a form of embodied difference can trigger personal and social consequences, from discrimination and stigmatization to support and collective action [34]. Social relationships help shape the experiences of women with disability. In contemporary society, women seek information and support through peer relationships in word-of-mouth informal communication, as well as in electronic formats mediated by the internet and known as electronic word of mouth (eWOM) [35]. In general, women, more than men, use social media to communicate with others, seek social support, and share personal experiences [36-38]. Both women with and without disability exchange advice, perspectives, and humor about pregnancy and motherhood through social media, including Web-based diaries or blogs. Personal blogs (Web-based diaries) are one type of eWOM that provides an asynchronous format allowing bloggers to communicate with many readers who can access the information at their convenience [39]. Blogs can also offer a format for peer-to-peer social media engagement in which users can give and receive support and information about disability and pregnancy. Bloggers, particularly women bloggers, tend to share personal experiences to seek social support or to simply share their life experiences [36-38]. By creating and sharing tacit and experiential knowledge, women create a public space for the expression and construction of their perspectives, values systems, and interactions with health care, disability, and pregnancy-related social entities [40-42].

### Objective

Women with intellectual disabilities report enjoying social media and having positive experiences using social media to develop friendships, form a social identity, and build self-esteem [43]. Women with physical disabilities who had been pregnant in the past decade cited the value of receiving information from other women who had experienced disability and pregnancy and the importance of seeking peer support [44]. As a research resource, blogs offer unsolicited first-person accounts in a naturalistic environment about pregnancy and disability topics that women choose to describe to a public audience. The purpose of this study was to examine blog content written by women who have experienced or have a desire to experience pregnancy and/or motherhood who are living with a spinal cord injury (SCI), traumatic brain injury (TBI), spina bifida, or autism. Our goal was to capitalize on the information contained in these blogs as a means of understanding the experience of pregnancy, delivery, postpartum recovery, and early motherhood among women with disability. Personal blogs have been examined using a systematic approach in other studies [45]. Although health blogs may be viewed as anecdotal information, aggregated data collected from multiple health blogs can provide insight into the public narrative about the intersection between reproduction and disability [46-48] and support the need for further focused research on topics of value to women with disability.

## Methods

### Methodology

Before beginning this systematic appraisal of blogs, an institutional review board (IRB) approval was sought and the study was acknowledged as nonhuman subject research through the University of Utah (IRB number 00105240). To maintain the privacy of individuals who published the blogs and were not individually consented for this research, we deidentified the content in the blogs and blog comments by anonymizing the details and quoted text before inclusion in this study to reduce the risk of bloggers being reidentified. To learn more about the perspective about pregnancy among individuals with disabilities, we selected 4 key types of disabilities as the target of our study. These included conditions resulting in impairments in movement and/or cognition and spanned developmental disabilities and acquired disabilities. Specifically, we selected SCI, TBI, spina bifida, and autism. We sought to include acquired and congenital conditions that impair either physical or cognitive abilities. Methods for the blog appraisal were similar to those reported in previous research [45].

### Blogs Selection for Study Inclusion

Using a consensus process, the research team decided upon keywords to use for blog searches. Personal blogs were systematically identified by searching Google using a specific search strategy for each keyword related to disability. Disabilities of interest included SCI, TBI, spina bifida, or autism. Both full terms and their acronyms (ie, SCI and TBI) were used in the search. We also used tetraplegia for SCI.

We developed a data extraction sheet containing the following inclusion criteria: (1) written by a woman or care partner (ie, parent or spouse) of a woman with a self-identified diagnosis of SCI, TBI, spina bifida, or autism, (2) focused on the personal experience of health and health care during the prepregnancy, prenatal, antepartum, intrapartum, and/or postpartum periods, (3) written in English, and (4) published between 2013 and 2017. There were no limitations on the blogger’s country of origin. Forums were omitted. The data extraction sheet was pilot-tested with 10 blogs and refined as needed to determine which blogs would be retained for analysis. Initially, 12,600 records were identified. A total of 296 blogs (eg, personal, news outlet, corporate, and foundation) were then identified. In the final step, 215 personal blogs were reviewed for inclusion by at least 2 independent reviewers (MLL and MJT). Unanimous agreement between at least 2 independent reviewers on the final blogs for inclusion was required. Using this method, the first 10 pages of each database search in Google were reviewed for the presence of eligible blogs by 3 of the authors (MLL, MJT, and SD). Exclusion criteria were applied to remove blogs that were duplicates and those that were not personal blogs. The remaining 125 personal blogs that met the inclusion and exclusion criteria were forwarded for qualitative descriptive analysis.

### Qualitative Descriptive and Thematic Blog Analysis

Using principles of qualitative description [49,50], the investigators developed and discussed inductive descriptive codes and code definitions. The first 15 blogs were initially read by 2 independent investigators (MLL and MJT), and the initial blog corpus for coding dictionary development was formed. Open coding of successive blogs using a line-by-line analysis was used to apply existing codes or develop new codes for segments of blog text using NVivo 11 software (QSR International) [49]. Codes were reviewed in a constant comparative process and clustered into categories representing similar constellations of blog content. Through a process of group analysis, all investigators then reviewed blog data, codes, and categories. Matrices organized codes by disability type stratified by reproductive health context (ie, preconception, prenatal, and postpartum) and resulted in the identification of overarching themes about women’s reproductive experiences and disability [51,52]. In the final analytic step, the research team reviewed the coded data again thematically, explicitly assessing blog text for content by disability type in the different reproductive health contexts.

## Results

### Overview

**Figure 1:**
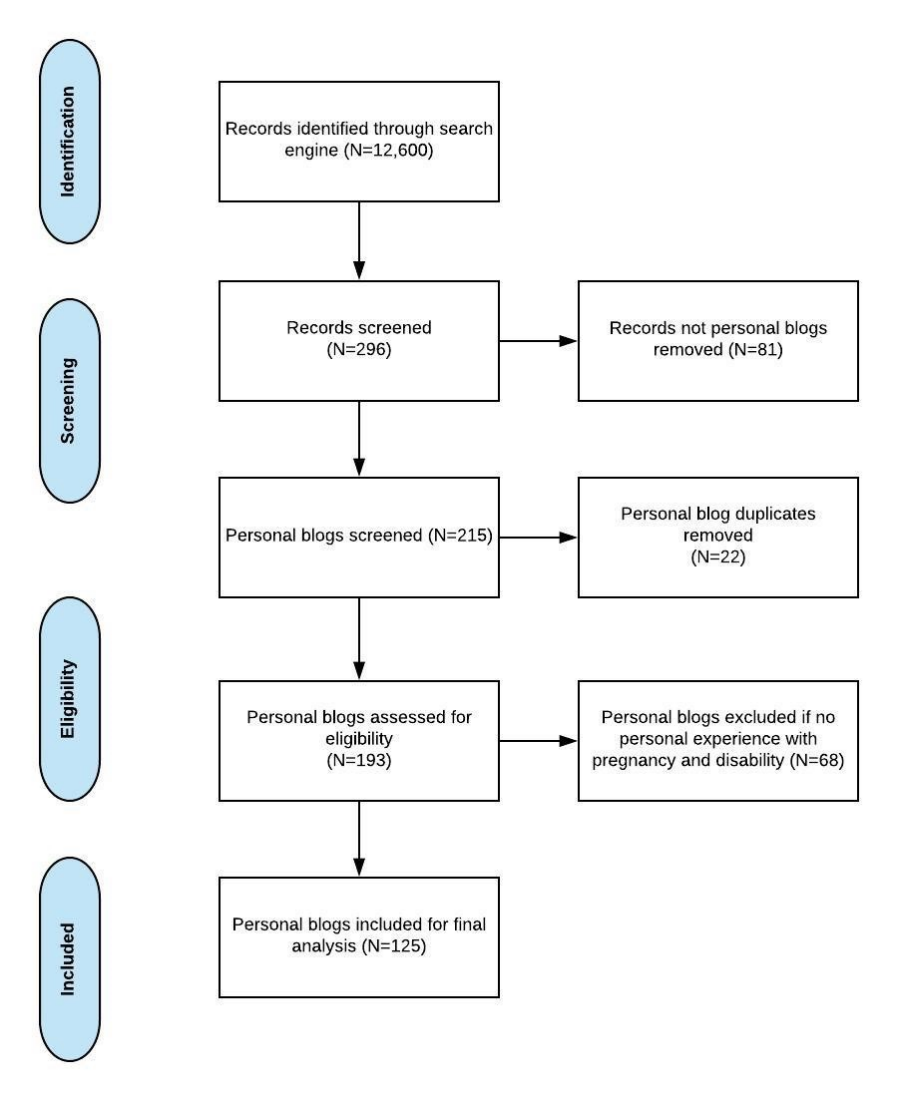
Preferred Reporting Items for Systematic Reviews and Meta-Analyses diagram.

Of the 296 website records identified, a total of 125 blogs met the inclusion criteria and were included in this qualitative analysis. Preferred Reporting of Items for Systematic Reviews and Meta-Analyses guidelines [53] were used to describe the blog inclusion process (see Figure 1). None of the identified blogs were written by a care partner; all were written by the women themselves. The majority of blogs analyzed were written by women with SCI and TBI. There were no blogs by women with spina bifida that were identified. There was 1 distinctive blog written by a woman who was diagnosed with autism following pregnancy. She reflected upon how her symptoms predated pregnancy and early motherhood, thus indicating a likelihood of her autism being present during pregnancy. Her situation was unique and clarified a different perspective of pregnancy and motherhood with a disability. It was not always overtly clear if the bloggers with TBI or autism used wheelchairs; however, all blogs written by women with SCI mentioned the use of wheelchairs. Of the blogs included, only 1 discussed abortion, 1 discussed miscarriage, and 3 mentioned breastfeeding (see Table 1 for additional blog characteristics).

In total, 4 key themes about the reproductive perspectives of women with disabilities resulted from the qualitative analysis: (1) (in)accessible motherhood, (2) (un)supportive others, (3) different, but not different; and 4) society questioning motherhood. How each theme was expressed across the reproductive contexts and by women with different disabilities is further described below. Themes by disability category and reproductive health context are described in Table 2.

### (In)accessible Motherhood

Women with disabilities face different challenges and barriers when considering or preparing for pregnancy. Adaptations were necessary to make motherhood more accessible because of the universal design of standard baby products. However, women showcased a sense of pride in successfully adapting baby products to realistically fulfill their motherhood needs.

**Table 1:** Blog Characteristics.

Blog characteristics		Disability type				Total
		TBI^a^	SCI^b^	Spina bifida^c^	Autism	
Blog posts, n (%)		37 (30)	83 (66)	—^d^	5 (4)	125 (100)
Blog post word count, average (range)		1185 (212-2057)	356 (202-1297)	—	925 (800-1612)	—
Blog posts with comments, n (%)		11 (50)	8 (36.4)	—	3 (13.6)	22 (100)
Women bloggers, n (%)		3 (12)	19 (76)	—	3 (12)	25 (100)
**Prepregnancy-related blog posts, n (%)**						
	Surrogacy	0 (0)	3 (100)	—	0 (0)	3 (100)
	Artificial insemination	0 (0)	1 (100)	—	0 (0)	1 (100)
	Invitro fertilization	1 (100)	0 (0)	—	0 (0)	1 (100)
Prenatal-related blog posts, n (%)		0 (0)	6 (100)	—	0 (0)	6 (100)
**Antepartum-related blog posts, n (%)**						
	Pregnancy	38 (50)	37 (48.7)	—	1 (1.3)	76 (100)
	Abortion	0 (0)	1 (100)	—	0 (0)	1 (100)
	Miscarriage	0(0)	1 (100)	—	0 (0)	1 (100)
**Intrapartum-related blog posts, n (%)**						
	Cesarean delivery	0 (0)	4 (100)	—	0 (0)	4 (100)
	Vaginal delivery	1 (16.7)	5 (83.3)	—	0 (0)	6 (100)
	Home birth	0 (0)	1 (100)	—	0 (0)	1 (100)
**Postpartum-related blog posts, n (%)**						
	Early motherhood	0 (0)	18 (100)	—	0 (0)	18 (100)
	Developmental milestones	0 (0)	18 (100)	—	0 (0)	18 (100)

^a^TBI: traumatic brain injury.

^b^SCI: spinal cord injury.

^c^No blog posts were identified for women living with spina bifida.

^d^N/A: not applicable.

#### Prepregnancy

Women in wheelchairs secondary to SCI reported emotional readiness to have a child but needed to consider the physical ramifications and potential adaptations. Surrogacy was discussed in 2 cases of SCI where the woman’s health status made pregnancy infeasible. In 1 case, discussions of surrogacy were complicated by the fact that Medicare (the federal health insurance in the United States for people with disabilities and older adults) does not cover the costs of surrogacy, resulting in 1 couple trying to sell their home and occasionally purchasing lottery tickets to achieve their reproductive health goals. In another case, the husband and wife were actively seeking surrogacy and were hopeful that they would become parents sometime soon.

In contrast, women with autism did not describe physical concerns related to pregnancy but expressed apprehension about their emotional capacity to care for a child. Concerns about difficulty coping with a newborn, such as changes in schedule and concerns about their ability to connect emotionally with their baby, were described.

#### Pregnancy

During pregnancy, some mothers reported a need for a larger wheelchair to accommodate the weight of their growing fetus. Pregnancy-related weight gain complicated transfers to and from the wheelchair, such as getting in and out of a car or going to the bathroom. In some cases, this reduced the woman’s independence. Some women experienced challenges with going uphill in a wheelchair during pregnancy because of the increased weight and changes to their center of balance; anti-tip bars were used as a safety net.

**Table 2:** Theme by reproductive health context and disability category.

Theme and reproductive health context		Blogs by disability category		
		SCI^a^	TBI^b^	Autism
**(In)accessible motherhood**				
	Prepregnancy	✓	—^c^	✓
	Pregnancy	✓	✓	—
	Delivery	—	—	—
	Early motherhood	✓	✓	—
**(Un)supportive others**				
	Prepregnancy	✓	✓	✓
	Pregnancy	✓	✓	—
	Delivery	—	—	—
	Early motherhood	✓	—	—
**Different, but not different**				
	Prepregnancy	✓	✓	✓
	Pregnancy	✓	✓	—
	Delivery	✓	—	—
	Early motherhood	✓	✓	✓
**Society questioning motherhood**				
	Prepregnancy	✓	—	✓
	Pregnancy	—	—	—
	Delivery	—	—	—
	Early motherhood	—	—	✓

^a^SCI: spinal cord injury.

^b^TBI: traumatic brain injury.

^c^Not applicable.

Medical exam tables were not always easily accessible during medical appointments. Women described needing their partners to help with transfers to and from medical exam tables, when such tables were not accessible. The majority of women with SCI described their maternity care provider as being inexperienced in caring for pregnant women with SCI, particularly during labor and delivery. Only 1 blogger discussed taking a prenatal class, noting that she had to make adaptations to what was taught to meet her own unique needs. The blogger shared that the class was tailored to childbearing mothers without disability and did not help to address her fears or provide relevant information about unique adaptations that would be necessary for her. She expressed interest in receiving tailored information about labor positions and newborn care and feeding for women with physical disability.

#### Early Motherhood

Mothers with disabilities reported using *life hacks* and practice to adequately manage their baby safely in an able-bodied world. Baby changing tables were lowered to accommodate those in a wheelchair. Some wheelchair-bound mothers reported practicing different techniques for holding the newborn while in a wheelchair, wheelchair mobility while holding a baby (given additional weight), transferring the baby, and best positions for comfort. Such practice was reported with the use of weighted dolls as well as without the dolls. One mother stated:

I can finally get the car seat in alone…I’m sure I would have been able to do it all along, we just never had the chance to “practice”! But I am soooo excited!

She also provided a 7-step process which included reclining the passenger seat down and twisting her body to face the back seat and placing the car seat in its base. To increase easy access to her child’s nursery, another mother redesigned her home, offering an accessible and comfortable environment to provide direct care to her baby when needed. Her blog highlighted how she planned the remodel and how the changes helped her to more easily care for her baby.

### (Un)supportive Others

Although most women reported consistent and positive support from family and friends during pregnancy and motherhood, the quality of support they received from health care providers was mixed. However, despite mixed feelings about health care provider support, women reported being proactive about their needs and perseverance to achieve with their pregnancy and motherhood.

#### Prepregnancy

All women bloggers who became pregnant reported doing so with a supportive partner. Family members, when mentioned, appeared to provide instrumental and emotional support. One woman stated:

I have a lot going for me. I already know what lots of people don’tparalyzed women (and me in particular) can conceive and deliver children normally. I have a supportive and loving husband who wants to have a child with me. I have many friends – some with greater problems than I havewho will be there for me. I have decades of experience working around my paralysis and playing to my strengths.

Some women delayed announcing their pregnancy to family and friends to prevent worry or because they were not sure how the pregnancy would be received. One woman described feeling more comfortable sharing her pregnancy news on the Web versus in person. In announcing her pregnancy, she celebrated the fact that by becoming pregnant, she was dispelling misperceptions that she was asexual or incapable of enjoying a sexual relationship, disconnecting pregnancy to motherhood.

In some instances, women were advised by their health care provider to abstain from having children and felt that their providers viewed women with disability as high-risk maternity patients. Women attributed this high-risk designation to health care providers’ lack of experience in caring for women with physical disabilities or to a biased view that women with disabilities should not have children. One woman with autism expressed, “Autistic people are people with their own wants and needs and goals, and we can make our decisions about what is and is not in our best interest.” Several bloggers reported interviewing multiple health care providers until they found one who supported their desire to become pregnant.

#### Pregnancy

Among women in wheelchairs, identifying offline and Web-based peers they could relate to offered them a sense of shared experience. In some cases, the desire to find a peer was the rationale for starting their blog. The individuals who commented on the blogs included both women with similar disabilities and others who could not be identified as having a disability or not. Comments from those who self-identified as having a disability indicated that reading about peers with similar experiences was validating. For example, a television program about a woman in a wheelchair who delivered a baby provided a sense of inspiration for some but caused worry for others, as the televised woman required bed rest.

Experiences with health care providers varied. Some women reported experiencing a shared decision-making process in planning for a cesarean or vaginal delivery. In some cases, women reported interviewing obstetricians either to determine those who had the most experience providing pregnancy care for women with their same disability or to find a health care provider with whom they felt the most comfortable. One mother stated:

I didn’t know any other moms in wheelchairs. I found an amazing doctor, but I was his first patient with a physical disability. We were going to learn the process together.

In contrast, some women reported having providers who were not supportive. Some women felt that their health care provider believed they should not be pregnant or would be unable to care for a child. One mother stated:

The unknown of paraplegic deliveries gets labeled as high-risk to doctors. Even though I didn’t feel I was high-risk, I heard their admonitions and dutifully went for a prenatal consultation with the most respected obstetrician I could find.

The proximity of the health care provider to the woman’s place of residence was also taken into consideration, as women wanted to be able to access health care quickly and easily if needed.

#### Early Motherhood

Some women relied heavily on others to support them throughout pregnancy, whereas other women who were previously independent considered accepting help from others for the first time during pregnancy. One woman stated:

The combined experiences of pregnancy and motherhood seemed to soften some lingering bitterness in me, and I became more grateful for help whenever it came my way. Help was not there all the time, however.

### Different, But Not Different

Although women with disabilities faced challenges related to their abilities, they reflected on their experiences and milestones as being similar to those experienced by women without disability. Furthermore, becoming pregnant as a woman with disability offered membership in a unique group and the bloggers expressed a sense of unity with their peers who shared this experience.

#### Prepregnancy

When expressing desires for pregnancy, women shared feelings of excitement but also reported apprehension about their ability to nurture a child and simultaneously manage their own disability. Some women had to stop medications before becoming pregnant whereas others worried about the potential need for bed rest. One woman indicated she was unsure if her paralysis would hinder her ability to push during delivery. Another woman considered surrogacy as a way to mitigate health risks, stating:

I thought one of the hardest parts of deciding surrogacy would be finding a compassionate man to support my choice. However, my husband was completely supportive of my decision, even while we were dating. When we became ready to start our family, we realized that surrogacy was going to be the only option for us.

Information-seeking behavior about reproductive health and pregnancy was common. Some women with autism highlighted the fact that those with autism are often very intelligent. One woman stated, “One theory I like is that we actually make great mothers because we research, research, research whenever we know we’re going to be encountering something new!” In a contrasting case, a woman who was diagnosed with autism after pregnancy indicated that she may not have been emotionally ready to raise a child, although she did not realize it at the time. The woman stated:

When you’re undiagnosed and going into parenthood, many issues can arise. However, becoming a parent when you know your disability can hinder your parenting abilities; this creates an entirely different dilemma.

Given the timing of her autism diagnosis, she did not have the opportunity to prepare for motherhood with an understanding of her autism diagnosis. The disability-specific experiences of women in the preconception context involved considering whether pregnancy was a good choice and whether they would be up for the perceived challenges of parenting with a disability. As women blogged about these considerations, they expressed emotions and concerns specific to their unique situations. Yet, some concerns were common among other women contemplating motherhood.

#### Pregnancy

Bloggers reported experiencing common pregnancy symptoms such as food cravings, heartburn, morning sickness, and fatigue. Bloggers also reported planning and participating in baby showers, shopping for baby items, and receiving baby gifts from friends and family. Pregnancy appeared to be relatively uneventful for some. One woman stated, “Throughout my pregnancy, I was still able to live completely independently.” Many women were told that they were categorized as *high-risk*, which may have increased the frequency of their medical appointments. However, the bloggers did not directly discuss the frequency of appointments. There was no mention in the blogs of women with SCI about common pregnancy complications in this population, including urinary tract infections, decubitus ulcers, constipation, increased spasticity, deep vein thrombosis, and alterations in pulmonary function [54,55]. In addition, no women with SCI mentioned increased urinary frequency or increased need for self-catheterization. However, 1 woman with SCI did offer advice on the need to stock up on supplies in case self-catheterization was needed.

There were multiple women with SCI who wrote about sadness or frustration when strangers were unaware of their being pregnant. They relished the opportunity to socially rejoice in their pregnancy but lamented the lack of notice. Some women wondered if strangers assumed that they had gained weight as a result of being in a wheelchair or felt that people had a hard time imagining that a woman in a wheelchair might possess the capability to conceive or be pregnant. One woman was direct about the perception that sexual intimacy and paralysis were incommensurate, indicating, “If I offer to share that I am pregnant with a stranger, the response is often a silent combination of confusion and disbelief.”

In a single case, 1 mother opted to have an abortion. Comments from blog readers without disability who had also undergone abortion indicated that the experience did not differ from others who had experienced an abortion, indicating feelings of isolation. A mother with SCI indicated that she had miscarried her second pregnancy near the second trimester expressed feelings of sadness, but mostly guilt because of her body’s betrayal and inability to sustain a full-term pregnancy. In this same instance, the woman shared that her partner reassured her by suggesting:

It just happens that way sometimes. The baby is weak and just doesn’t thrive and that’s all it is.

Women with disability may seek out abortion, experience miscarriage, and have a variety of common emotions after these experiences.

#### Delivery

Most women perceived that their delivery was *normal*. Some women with SCI reported experiencing a vaginal delivery with a short labor and tolerable pain because of paralysis, which was viewed positively. For those with SCI who had some lower abdominal sensation, an epidural was pursued; although, at times, it was challenging to insert because of spinal hardware. One woman indicated:

Although I cannot feel pain below my injury site, my body is experiencing it all. Due to this, I was given an epidural to help my body endure the pain after childbirth even though I knew I wouldn’t feel a thing.

In a singular case, 1 woman had an uncomplicated delivery at home with a midwife and doula and reported a good experience. Other women had planned cesarean deliveries, though no one individual mentioned multiple cesarean deliveries. There were no reports of planned vaginal deliveries that resulted in cesarean delivery. However, 1 woman indicated a shared decision-making process between herself and her obstetrician resulting in the decision to pursue a planned cesarean delivery owing to her inability to push because of the titanium rods in her spine. There were no mentions of in-patient lengths of stay; however, 1 woman was required to be on bed rest following delivery because of hypotension. Furthermore, some women indicated that the utilization of intrapartum anesthesia required some adjustments on the basis of SCI, and the women and their health care providers problem solved these challenges, including the unusual decision in 1 case to administer general anesthesia during delivery. Another woman experienced an adverse event, a spike in temperature and uncontrollable shaking, which she attributed to her body releasing more Oxytocin than normal to compensate for her paraplegia. There were no specific details about women with autism or TBI and their labor or delivery.

#### Early Motherhood

All bloggers indicated that they were able to provide direct newborn care and celebrated their baby’s milestones. Breastfeeding was mentioned by 3 different bloggers; it is unknown if the other women were breastfeeding or not. Of the 3 women who did breastfeed, 1 indicated that she did not experience challenges with breastfeeding, although she did report the use of pillow props to support positioning, a technique also used by women without disability. There were no mentions of not to breastfeed because of disability or disability-related challenges. The blogs of women with autism or TBI contained no mention of breastfeeding at all. Additionally, there were no mentions of use of a breast pump, mastitis, or breastfeeding problems in any of the blogs.

Mothers used both unique and standard ways to carry their child while in a wheelchair. For example, 1 mother used an airplane seatbelt to attach a baby basket carrier to her wheelchair whereas other mothers used baby carriers. One mother wrote:

My greatest ally in managing a newborn on my own was a baby sling. Being in a chair and trying to carry a baby felt like having both arms and both legs tied behind my back. The baby sling freed up my arms and allowed me to work and do other things while holding the baby. I learned how to do almost anything wearing my sling, including using the toilet.

Women also blogged about the challenges of transitioning to motherhood, reflecting on challenges they believed that they shared with other mothers. One woman with autism emphasized how the transition to motherhood may be particularly challenging for those with autism, writing:

Babies have demanding round-the-clock needs; they are everything that an autistic person would have difficulty coping with. Time alone and carefully crafted routines no longer existed. My body was no longer my own; transformed first by pregnancy then by postpartum hormones and breastfeeding.

Schedule challenges are likely not unique to women with autism but perhaps may be more disruptive to women with autism who adhere to routines to manage their needs. We found no mentions about postpartum depression or depressive symptoms in any of the blogs reviewed.

### Society Questioning Motherhood

Women experienced misconceptions or misunderstandings from others, including questioning whether or not a woman with a disability should conceive, deliver, and care for children. Yet, women refused to give up the opportunity to experience motherhood.

#### Prepregnancy

Some women blogged about others questioning their ability to be or become a mother. For those in wheelchairs, this was often rooted in misconceptions about whether or not they were infertile or could and/or would engage in sexual intercourse. One woman, in an attempt to reconstruct social norms, stated “With adaptations, many moms with paralysis are capable of raising children.” Other women in wheelchairs indicated that they would not let society get in their way of becoming mothers. One woman stated:

One day, when our children are old enough, we will tell them the story of the nonconventional delivery, and we will share how hard we—their father and I—worked to make their existence possible. Because that’s what parents do: they find a way to make possible the impossible.

On the contrary, 1 blogger with autism wrote about ongoing social resistance to parenthood targeting people with disabilities. Referencing the historical record of involuntary sterilization of women with disabilities, she indicated that she would be fearful if she publicly announced she had autism and was pregnant because of the backlash she would receive.

#### Early Motherhood

Although questions and concerns about dealing with the challenges of motherhood were discussed, many women expressed their excitement about being a mother and gratitude for their supportive partners. A concern about public scrutiny of the emotional and physical abilities of women with autism to care for a child was noted by 1 blogger with autism, who wrote:

The biggest issue occurs when people in authority misinterpret us [women with autism] and call in Child Protective Services, when in reality, nothing is wrong. Ultimately, we are individuals, influenced by being autistic, influenced by being female, but in the end, still individuals.

Women blogged about how society expected them to face emotional or physical challenges during pregnancy and motherhood; however, the women themselves expressed overall feelings of capability in bearing and caring for children.

## Discussion

### Principal Findings

In this study, we sought to learn more about first-person publicly available accounts of prepregnancy, pregnancy, delivery, postpartum recovery, and early motherhood as written in the blogs of women with SCI, TBI, spina bifida, or autism. We examined personal stories about encounters with the health care system as well as the ways women used blogging to convey information and offer support to other women with disability and their families. Blogs represent an important piece of the public narrative about the intersection between disability, reproduction, and motherhood. Although we selected 4 key types of disabilities as the focus of our work to represent both acquired and congenital disabilities, our systematic search of personal blogs resulted in locating more blogs written by women with SCI and TBI than those with autism. We identified no blogs written by women with spina bifida meeting our search criteria.

In contrast to results from other studies that highlight the challenges faced by women with disabilities in achieving their reproductive goals [56-58], some of the blogs analyzed in this study included a more positive representation of women’s experiences. Although women wrote about negative interactions with providers, we also identified bloggers who felt that their experiences were empowering and who wrote about self-advocacy to achieve their reproductive goals with healthy outcomes. Across blogs, we identified numerous women with disability who had strong desires for motherhood, who had the support to pursue these desires, and who were able to successfully navigate a health system in pursuit of pregnancy. Women wrote that their disability did not compromise their pregnancy experience. Women even mentioned some ways in which their disabilities contributed to better experiences, such as the inability to feel pain during labor for some women with SCI or the structured, organized approach of women with autism as a positive attribute of parenting. Of note, most women seemed to be navigating health care for pregnancy and birth without formal collaboration between their maternity health care providers and their disability-specific providers.

Results derived from this blog analysis offer guarded yet positive counterpoints to the recursive cascades of disability, poor health outcomes, and insufficient social and structural support [59]. We suggest 3 possible interpretations of these results juxtaposed against the premise of syndemics theory that social, biological, and structural factors increase the risk for compromised reproductive health outcomes by reinforcing disadvantage [15,16]. First, the altruism of bloggers in paying forward their experiences to benefit others is an effort to build social capital within the reproductive-aged community of women with disability. Similar efforts identified in other social media health research [60] are unaccounted for in the syndemics theory. In this analysis, bloggers noted difficulties and challenges in their reproductive health choices and outcomes. Despite describing some negative experiences, women addressed how they navigated care using self-advocacy and social support to achieve desired outcomes. Overall, the blogs went beyond simply describing difficulties, as bloggers detailed the ways in which their choices and *life hacks* could benefit other women in similar situations.

Second, social media posts tend toward positive self-representations [61,62], a phenomenon that could mute the presentation of difficult experiences or poor reproductive health outcomes fueled by the interaction between disability and disadvantage. Selective overrepresentation of positive experiences in personal blogs could possibly account, to an unknown degree, for our findings. Yet, the value and uniqueness of blogs also argues for their inclusion in research for several reasons. Blogs allow for detailed and complete stories to be told. Blogs have been used previously to learn more about how individuals share clinical data with family and friends [45], the caregiving perspective [63], and the lived experience of having a chronic condition [64]. Blogs humanize the experience of reproductive health by providing access to the positive experiences, joys, and achievements of women with disability to counterbalance the negativity of published medical research or health care providers. As some suggest, blogs put the *public* back into public health [65]. This rich source of data can be used in addition to or as a precursor to qualitative interviewing.

A third explanation for the generally positive pregnancy and disability spin in blogs is that online social support transmitted through the blogging endeavor and eWOM may offset the isolation and social disadvantage that women with disability experience. This social support effect has been documented among adults with chronic pain [66], a population with similar situational disadvantages. Social media platforms can contribute and facilitate knowledge sharing [41,42]. By analyzing those personal experiences, we identified several unmet needs of women with SCI, TBI, and autism, such as a need for more accessible exam tables, a need for larger wheelchairs as pregnancy advances, and a need for supportive health care providers who respect women’s choices to make autonomous decisions about whether to have children. Women bloggers offered suggestions and possible solutions to some common challenges. For example, several life hacks offered tacit knowledge to a community of women who may have similar disabilities or challenges in pregnancy and early motherhood. Although the tacit knowledge or personal experience is informal and lacks a robust evidence base, these suggestions may provide a starting point for further research and potential integration into the care of women with disability.

Use of blogs for research purposes has some limitations. For example, bloggers may comprise an atypical sample of the population of women with SCI, TBI, or autism. Bloggers are likely to have higher levels of literacy, be more technologically adept, and have higher levels of education [47,67]. Given the nature of our data collection methods, we were not able to extract demographic factors from bloggers beyond their self-identified disability types. Reviewing blogs clearly does not provide a comprehensive overview of the experiences and challenges faced by women with disability. Thus, our findings should be interpreted in this context. All bloggers in our study who mentioned partners described that they were supportive. It is important to note that none of the 5 blogs written by women with autism mentioned partners in their blog. We did not identify (1) financial concerns such as the increased medical costs for a high-risk pregnancy for the woman or infant, except for surrogacy; (2) lack of access to medical providers, although some struggled to find the right provider for them; (3) significant complications experienced by the mother or infant throughout pregnancy or delivery; or (4) postpartum depression. These concerns, commonly identified in the research about pregnancy and disability, were absent in this sample. For example, autonomic dysreflexia is a major concern during pregnancy and labor among women with SCI, particularly those with lesions above the T6 level [55]. However, we did not observe any specific references to autonomic dysreflexia among women with SCI in our blog analysis. Similarly, postpartum depression is often identified in women with autism but was not identified in the blogs of autistic women reviewed for this study [68]. In addition, our findings may not be generalizable to women with disability who do not write or read blogs, women with more complex medical histories, or women with disabilities other than SCI, TBI, and autism. Our search strategy did not identify blogs written by women with spina bifida and identified very few blogs by women with autism. It is possible that our search strategy did not identify all possible blogs. Alternatively, it is also possible that women with spina bifida and autism are not blogging to the same extent as women with SCI or TBI.

Application of findings from this study may advance research and practice. Women with disability who have been able to achieve pregnancy and motherhood have much to share with their peers who desire this experience. Using women with disabilities as health navigators has been recommended by other authors who have suggested that these navigators could help others to recognize their own potential for giving birth, understand the importance of advocacy and support during pregnancy and birth, share information, provide guidance on interacting with maternity providers, and help women manage their fears [69]. Future research may specify survey and interview questions on the basis of what women with disability highlighted as important personal and social resources for successful pregnancy and motherhood and challenges they encountered. Research is warranted to explore how other social media tools are being used in this context or could be utilized to help enhance information sharing and social support among women with disability. Some informational resources exist for some types of disability but for others, more information and resources are needed. Having all pregnancy-related resources in one Web-based location would be beneficial for both women with disabilities and the health care providers who care for them. Finally, tools to help women communicate with their providers about their unique needs and challenges would help to improve their health care experience from preconception through early motherhood. Such tools need to be developed and evaluated.

### Conclusions

This study of personal blogs written by women with SCI, TBI, and autism about pregnancy and health care offers a glimpse into their reproductive experiences, challenges, and adaptations and provides a foundation for future research to address the unique needs of women with disabilities.

## Abbreviations

eWOM: electronic word of mouth
IRB: institutional review board
SCI: spinal cord injury
TBI: traumatic brain injury
